# Comparison of effectiveness between warm acupuncture with local-distal points combination and local distribution points combination in breast cancer-related lymphedema patients: a study protocol for a multicenter, randomized, controlled clinical trial

**DOI:** 10.1186/s13063-019-3491-4

**Published:** 2019-07-05

**Authors:** Chien-Hung Yeh, Tian Yi Zhao, Mei Dan Zhao, Yue Wu, Yong Ming Guo, Zhan Yu Pan, Ren Wei Dong, Bo Chen, Bin Wang, Jing Rong Wen, Dan Li, Yi Guo, Xing Fang Pan

**Affiliations:** 1 College of Acupuncture and Massage, Tianjin University of Traditional Chinese Medicine, No. 312, Anshan West Road, Nankai District, Tianjin, 300193 China; 2 Department of Combined Chinese & Western Medicine, Tianjin Medical University Cancer Institute & Hospital, National Clinical Research Center of Cancer, Tianjin, China; 3 Department of Traditional Chinese Medicine, Tianjin University of Traditional Chinese Medicine, Tianjin, China; 4 Acupuncture Research Center of Tianjin University of Traditional Chinese Medicine, Tianjin, China

**Keywords:** Acupuncture, Lymphedema, Breast cancer

## Abstract

**Background:**

Lymphedema is the most common complication after breast cancer treatment, but management of lymphedema remains a clinical challenge. Several studies have reported the beneficial effect of acupuncture for treating breast cancer-related lymphedema (BCRL). Our objective is to verify the effectiveness of warm acupuncture on BCRL and compare the effectiveness of a local distribution acupoint combination with a local-distal acupoint combination for BCRL.

**Methods:**

This is a study protocol for a multicenter, three-arm parallel, assessor blinded, randomized controlled trial. A total of 108 participants diagnosed as BCRL will be randomly allocated in equal proportions to a local distribution acupoint (LA) group, a local-distal acupoint (LDA) group, or a waiting-list (WL) group. The LA and LDA groups will receive 20 acupuncture treatment over 8 weeks with local distribution acupoint combination and local-distal acupoint combination, respectively. The WL group will receive acupuncture treatment after the study is concluded. The primary outcome is the mean change in inter-limb circumference difference from baseline to week 8. The secondary outcomes include volume measurement, skin hardness, common terminology criteria for adverse events 4.03 (edema limbs criteria), stages of lymphedema from the International Society of Lymphology, Disabilities of the Arm, Shoulder and Hand questionnaire, and the Medical Outcome Study 36-item Short-form Health Survey.

**Discussion:**

This study aims to provide data on warm acupuncture as an effective treatment for BCRL and at the same time compare the effectiveness of different acupoint combinations.

**Trial registration:**

ClinicalTrials.gov: Identifier NCT03373474. Registered on 14th December 2017.

**Electronic supplementary material:**

The online version of this article (10.1186/s13063-019-3491-4) contains supplementary material, which is available to authorized users.

## Background

Lymphedema is the most common complication after breast cancer treatment, with an average incidence rate of 21% [1]. Some of the symptoms associated with breast cancer-related lymphedema (BCRL) are discomfort, pain, heaviness, tightness, stiffness, weakness, and a decreased range of motion in the affected arm, which can cause severe physical morbidity. The swollen appearance of the arm can also cause psychological distress, such as depression and anxiety, since it is a constant reminder of breast cancer [2].

Although several treatment options are available, including manual lymphatic drainage, compression bandaging, and complete decongestive therapy, a clinical guideline on the integrative therapies used after breast cancer treatment reported that there were no A-graded or B-graded therapies to report for lymphedema [3]. Therefore, the treatment of lymphedema is difficult and probably requires multi-disciplinary attention. In several National Comprehensive Cancer Network guidelines, acupuncture is recommended for the supportive care of cancer to reduce symptoms and side effects of conventional cancer care [4]. Recent studies have begun to investigate the therapeutic effect of acupuncture on BCRL and results showed the potential of acupuncture treatment for BCRL [5–10]. However, previous studies were mostly pilot and observational studies with small sample sizes and, thus, the effectiveness of acupuncture needs to be further confirmed with a larger trial. In addition, no consensus has been reached on the choice of acupoints to achieve optimal results. For example, Cassileth et al. [8] performed a whole-body treatment that included acupoints on the abdomen, legs, and affected and unaffected arms, while Yao et al. [5] performed acupuncture treatment on the affected arm only. Although both acupuncture prescriptions were able to reduce arm circumference, which of the two provides better results remains unclear. Therefore, we propose a multi-center, randomized controlled trial to determine the optimal acupoint combination for the treatment of BCRL.

In this study, we aim to determine the effectiveness of warm acupuncture in the treatment of BCRL with a rigorous, larger, multicenter, randomized controlled trial. In addition, we will compare the effectiveness between different acupoint combinations. Specifically, we aim to determine whether a local-distal acupoint combination or a local distribution acupoint combination is more effective in the treatment of BCRL.

## Methods

### Study design

**Fig. 1:**
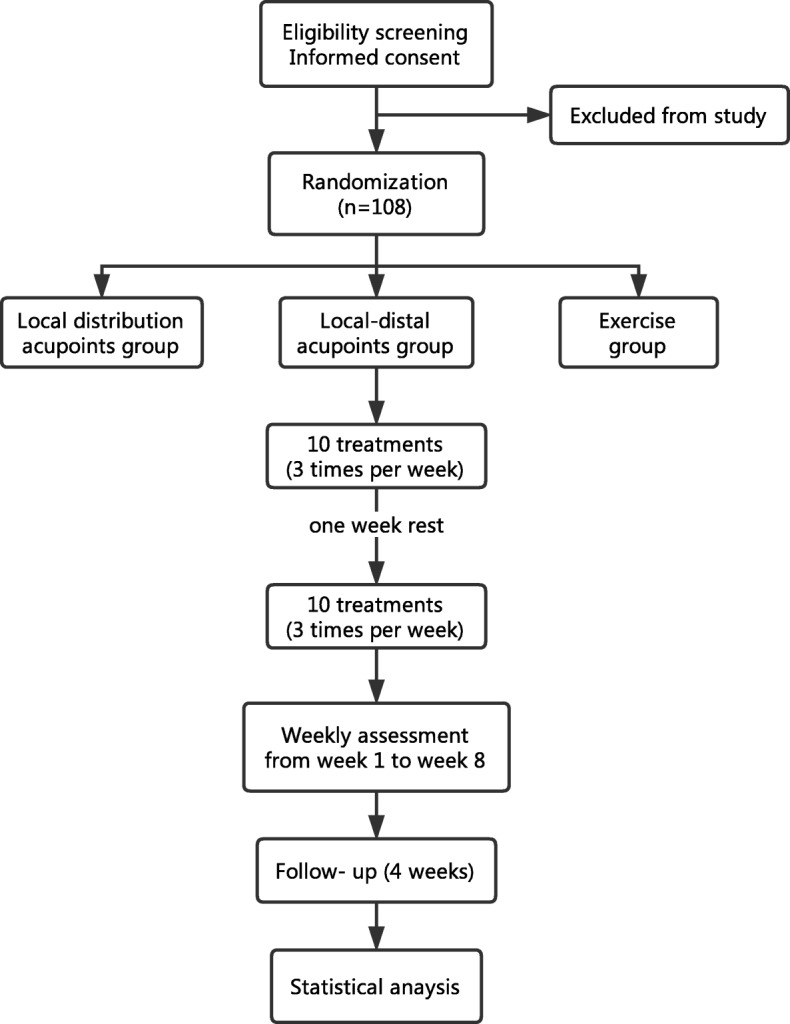
Study flow chart

This study is a multicenter, three-arm parallel, assessor blinded, randomized controlled trial conducted in China. A total of 108 patients will be randomly assigned to local acupoint (LA) group, a local-distal acupoint (LDA) group, or a waiting-list (WL) group in a 1:1:1 ratio. The schedule of enrolment, interventions, and assessments is summarized in Table 1, and the study flow chart is shown in Fig. 1. Standard Protocol Items: Recommendations for Interventional Trials (SPIRIT) 2013 Checklist is attached as Additional file 1.

**Table 1:**
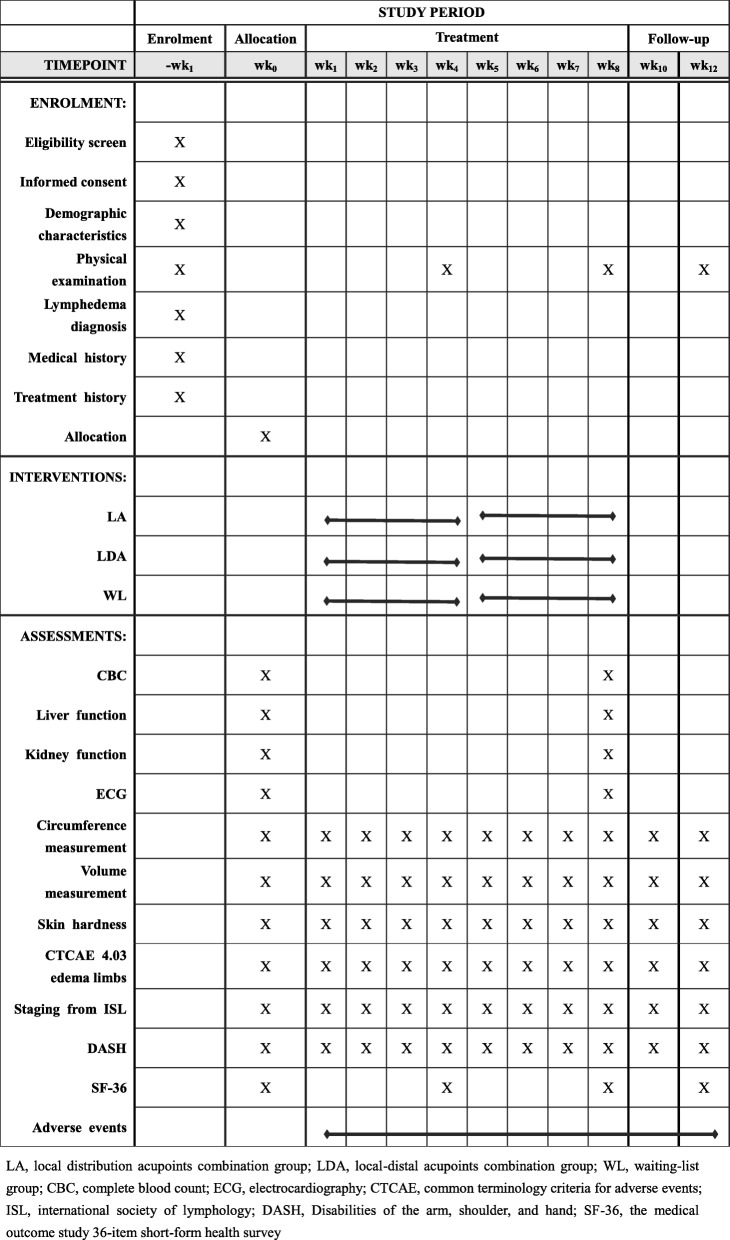
Schedule of enrolment, interventions and assessments

LA, local distribution acupoints combination group; LDA, local-distal acupoints combination group; WL, waiting-list group; CBC, complete blood count; ECG, electrocardiography; CTCAE, Common Terminology Criteria for Adverse Events; ISL, International Society of Lymphology; DASH, Disabilities of the arm, shoulder, and hand; SF-36, the Medical Outcome Study 36-item Short-Form Health Survey

### Recruitment

Participants will be recruited from the Tianjin Medical University Cancer Institute and Hospital, Baokang Hospital Affiliated to Tianjin University of Traditional Chinese Medicine, The Second Affiliated Hospital of Baotou Medical College, Henan Cancer Hospital, Gansu Provincial Cancer Hospital, and Sichuan Cancer Hospital by billboard advertisement and practitioner referrals.

### Inclusion criteria

1. At least 6 months after breast cancer surgery and with persistent breast cancer-related upper extremity lymphedema for at least 3 months. Upper extremity lymphedema is defined as more than 2 cm circumference difference or 5% volume difference between the affected and unaffected arms.
2. Presence of stage II or III lymphedema according to the 2016 consensus by the International Society of Lymphology [11].
3. A Karnofsky Performance Score ≥70
4. Men or women aged 18–80 years
5. Out-patients
6. Estimated life expectancy >6 months

### Exclusion criteria

1. Bilateral BCRL
2. Taking diuretics
3. History of primary lymphedema
4. A diagnosis of severe heart, liver, kidney, or hematologic disease
5. Edema caused by upper extremity disability or other conditions such as heart failure, kidney disease or malnutrition
6. Edema caused by recurrent or metastatic breast cancer
7. Hypoproteinemia
8. Inflammation, scar, or trauma at the site of operation, or other active skin infections
9. Unable to self-care, history of psychological disorders, or unable to communicate
10. Received lymphedema treatment within the past 1 month
11. Pregnancy or breastfeeding
12. The presence of electronic medical device implants
13. Denial to sign the informed written consent or unwillingness to conform to randomization
14. Participation in other clinical trials during the study period

### Randomization and allocation concealment

After signing the informed consent, eligible participants will be randomly assigned to one of the three groups by center randomization. The Clinical Evaluation Center at the China Academy of Chinese Medical Science in Beijing will be responsible for the generation of a random number and group assignment, which will be provided through the website at http://118.144.35.11/crivrs/index.htm. The practitioner who will perform the acupuncture treatment will then assign the participant to that intervention.

### Blinding

The assessor who will collect the data and the statistician who will perform the statistical analysis will be blinded to group assignment. Acupuncturist blinding cannot be achieved due to the nature of the intervention. Participant blinding is limited to the LA group and LDA group, since the WL group cannot help but notice their allocation.

### Interventions

The acupuncture groups (LA and LDA group) will receive acupuncture treatments three times per week for a total of 20 treatments. The treatments will be performed by practitioners who hold a Chinese medicine practitioner license from the Ministry of Health of the People’s Republic of China. Practitioners will be instructed to achieve de qi sensation and then the needles will be retained for 30 min. Disposable, sterilized stainless-steel acupuncture needles will be used in the acupuncture groups (Huatuo disposable acupuncture needle, Suzhou Medical Co., Jiangsu, China, 0.25 × 40 mm). During the needle retention period, moxa cones (Mac mini needle moxa, Tianjin HaingLimSouWon Medical Co., Ltd., Tianjin, China, 2 cm) will be placed on the handle of the needles at specific acupoints. A piece of hard paper will be placed on the acupoint to prevent burning of the participants from falling ashes. The moxa cones will then be burned to delivery warm acupuncture. Additional hard paper will be added if the patient feels uncomfortable with the increasing temperature. All treatment that may affect the result of the study will be restricted, including surgical interventions, acupuncture, blood-letting, diuretics, exercise, complete decongestive therapy, compression therapy, and manual lymphatic drainage. Treatments that the participants had been using prior to the trial may be allowed at the discretion of the investigator after evaluation of the patient’s condition.

### Acupoint prescription set

Local points set: Waiguan (TE5), Quchi (LI11), Sidu (TE9), Shaohai (HT3), Naohui (TE13), and Xiajiquan on the affected arm.

Additional local points set: Chize (LU5), Quze (PC3), Zhizheng (SI7), Yangchi (TE4), Zhongzhu (TE3), Qingling (HT2), Tianjing (TE10), Jianyu (LI15), and two other points according to symptoms on the affected arm.

Distal points set: Waiguan (TE5), Quchi (LI11), Shaohai (HT3), Xiajiquan on the unaffected arm; Guanyuan (CV4), Qihai (CV6), Shuifen (CV9), Zhongwan (CV12), bilateral Sanyinjiao (SP6), and Yinlingquan (SP9).

**Fig. 2:**
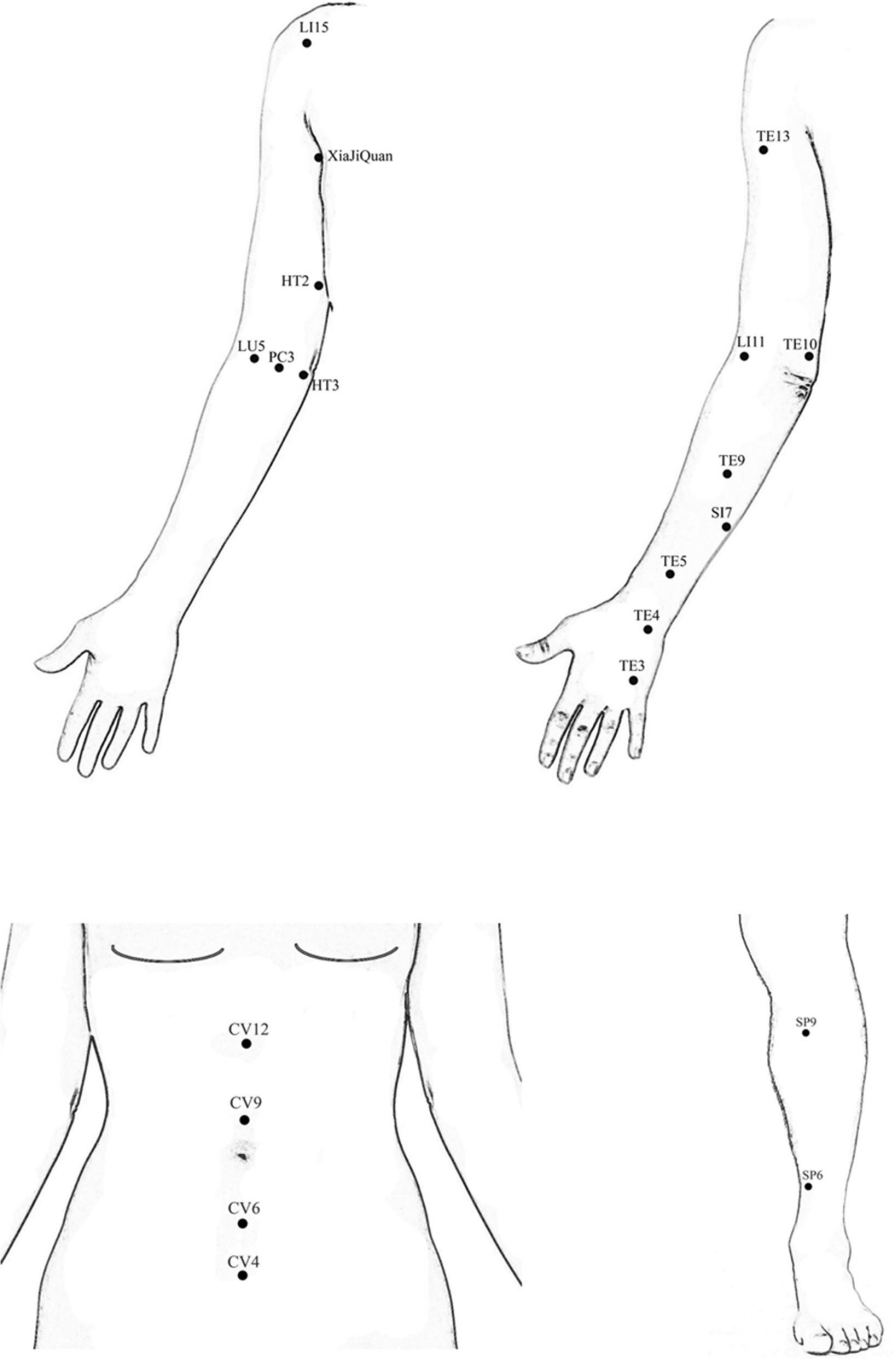
Acupuncture points used in the study

Detailed location of the acupoints are shown in Fig. 2.

### LA group

Participants will receive acupuncture treatment using the local points set plus the additional local points set (local distribution points combination). Warm acupuncture will be applied at Naohui (TE13), Quchi (LI11), and Sidu (TE9) if permitted, and one other acupoint according to the symptom on the affected arm.

### LDA group

Participants will receive acupuncture treatment using the local points set plus the distal points set (local-distal combination). Warm acupuncture will be applied similarly to the LA group, with the addition of Qihai (CV6), Shuifen (CV9), bilateral Yinlingquan (SP9), and Quchi (LI11) on the unaffected arm.

### WL group

Patients in the WL group will not receive any acupuncture treatment during the study. However, for ethical consideration, 20 free acupuncture treatments will be offered after the study is completed.

### Outcomes

#### Primary outcome measures

Various assessment methods are available, but circumference measurement is simple, convenient, low cost, and reliable [12]. Therefore, the primary outcome measures will be the mean change in inter-limb circumference difference from baseline to the end of the 8-week intervention. The circumference will be measured using a measurement tape (Hoechstmass Balzer Gmbh, Sulzbach,Germany) at the wrist crease, 10 cm above the wrist crease, elbow crease, 10 cm above the elbow crease, where the lymphedema is most severe, and at its corresponding location on the unaffected limb. The circumference difference will be assessed at baseline and before intervention at weeks 1–8.

#### Secondary outcome measures

Volume measurement is also commonly used for the evaluation of lymphedema and the mean change in inter-limb volume difference from baseline to the end of the 8-week intervention will be included as the second primary outcome measure. The volume of the affected and unaffected limbs will be measured by the volumetric measuring device (Baseline, USA) using the water displacement method, which is considered as the most reliable method for volume measurements [13]. The volume difference will be assessed at baseline and before intervention at weeks 1–8.

Skin hardness will be measured at places where the skin feels most tense to the touch by the NSCING SHORE LX-A durometer (Nanjing SuCe Measuring Instrument Co., Ltd., Nanjing, China). Skin changes such as increased tissue resistance and skin elasticity are often found in lymphedematous skin [14]. Our preliminary study found that patients felt more comfortable and less tense in the affected limb after acupuncture treatments. Therefore, we will use the muscle hardness tester to evaluate the effect of acupuncture on soft tissue tension. The change in skin hardness will be assessed at baseline and before intervention at weeks 1–8.

The Common Terminology Criteria for Adverse Events (CTCAE 4.03) [15] will be used to grade the severity of swelling using the edema limbs criteria. A grading of mild, moderate or severe swelling will be assessed based on the inter-limb circumference or volume discrepancy, anatomic architecture, appearance, or activities of daily living. The CTCAE 4.03 will allow us to evaluate the clinical significance of circumference change. The CTCAE 4.03 edema limb grading will be assessed at baseline and before intervention at weeks 1–8.

Stages of lymphedema from the International Society of Lymphology will be used to grade the severity of lymphedema [11]. Staging of 0, I, II, or III will be assessed based on severity of swelling, ability to reduce swelling by elevation, and skin changes. Staging of lymphedema will be assessed at baseline and before intervention at weeks 1–8.

The Disabilities of the Arm, Shoulder and Hand (DASH) questionnaire is a scale that consists of two concepts – functional status (part A) and symptoms (part B). The functional status part is further divided into three dimensions, namely physical, social, and psychological. The total score of the DASH ranges from 0 to 100, with higher scores representing worse symptoms and function. The DASH has good validity and responsiveness and it is recommended to assess upper extremity function in breast cancer survivors [16]. The validated Chinese version of the DASH will be used in this study [17]. The DASH will be assessed at baseline and before intervention at weeks 1–8.

The Medical Outcome Study 36-item Short-Form Health Survey (SF-36) is a commonly used instrument to assess quality of life and has good validity [18]. The SF-36 includes the following eight concepts: physical functioning, role limitations due to physical problems, social functioning, bodily pain, general mental health, role limitations due to emotional problems, vitality, and general health perception [19]. The validated Chinese version of the SF-36 will be used in this study [20]. The SF-36 will be assessed at baseline and before intervention at weeks 4 and 8.

### Safety assessment

All adverse event occurrences will be informed immediately to the clinical research coordinator and the principle investigator. Together with the acupuncturist in charge, they will evaluate, consult on the case, and take proper action. All expected (feeling faint after acupuncture treatment, stuck needles, broken needles, minor burning and vesicles during or after warm acupuncture, hematoma and bruising after needle removal) and unexpected (exacerbation of lymphedema, inflammation, infection, local or systematic reaction) adverse events will be reported immediately and recorded in the case report form (CRF). Any significant change of health state after baseline will also be recorded as an adverse event. All adverse events will be closely monitored and followed up until stabilization or resolution. Complete blood count, liver function, kidney function, and electrocardiography will be assessed at baseline and at week 8 to detect adverse events.

### Sample size calculation

According to the results of our preliminary trial (9 participants in the LDA group, 7 participants in the LA group, and 10 participants in the WL group), the biggest circumference difference after 20 treatments was 2.34 ± 1.6 cm, 3.32 ± 1.53 cm, and 3.74 ± 1.1 cm in the LDA, LA, and WL groups, respectively. According to the formula:

$$ \mathrm{n}={\Psi}^2\left(\sum \left({Si}^2\right)/K\right)/\left[\sum {\left(\overline{Xi}-\overline{X}\right)}^2/\left(\mathrm{K}-1\right)\right] $$

1. α = 0.05
2. β = 0.10
3. K = 3
4. Ψ: K = 3, degree of freedom V1 = K–1 = 2; degree of freedom V2 = N–1, N is unknown, assume N as ∞, according to the T distribution critical values table when α = 0.05 and β = 0.10: Ψ α, β, K–1, ∞ = 2.52
5. $\overline{Xi}$ and Si represent mean number (X1 = 2.34, X2 = 3.32, X3 = 3.74) and standard deviation (S1 = 1.6, S2 = 1.53, S3 = 1.1) of group i according to the preliminary trial.
6. $$ \overline{\mathrm{X}} $$= (X1 + X2 + X3)/K = (2.34 + 3.32 + 3.74)/3 = 3.13

The result of calculation was 30 participants in each group. Assuming a 20% dropout rate, a total of 108 participants are required with 36 participants in each group.

### Statistical analysis

A full analysis set will include all randomized participants who received at least one treatment and one follow-up. The principle of the last observation carried forward will be used in the case of missing data. The number of end point evaluations in each group will be kept the same as the number of participants in each group at the beginning of the trial.

A per-protocol set will include the following: (1) participants who meet the criteria of the protocol, (2) participants with measurable primary outcomes, and (3) participants without major violation of the protocol.

The statistical analysis of the primary outcome will be analyzed with a full analysis set and per-protocol set separately. The safety set will include all randomized participants who received at least one treatment.

Data will be coded and entered into SPSS (v.22) for statistical analysis. The Cochran–Mantel–Haenszel test will be used for analysis of center effect. Descriptive statistics of all sociodemographic and clinical data will be included. Continuous variables will be reported using mean and SD for normally distributed data or median and range for skewed data. Categorical variables will be expressed as number and percentage. For outcome measures, the mean differences from baseline values to the end of treatment will be compared using ANCOVA. Repeated measures analysis of variance (R-ANOVA) will be used to assess the inter-limb circumference difference, inter-limb volume difference and skin hardness between the three study groups. Inter-group differences in categorical data (CTCAE 4.03, stages of lymphedema) will be assessed using the χ^2^ test or Fisher’s exact tests (two-tailed), as appropriate. Linear mixed models (for continuous outcome variables) and generalized estimating equations (for categorical outcome variables) will be used to examine the change of intensity in inter-limb circumference difference and SF-36. *P* values of <0.05 will be considered statistically significant.

### Patient and public involvement

Patients and public were not involved in the development of this protocol.

### Data collection and management

To warrant quality of the data, a well-trained assessor will be responsible for data collection and recording on the CRF. Double entry of the data into the online trial database will be implemented by clinical research coordinators. Regular monitoring of the recruitment, intervention and assessment processes will be performed to ensure predetermined protocol and standard operating procedures are followed. All CRFs will be stored in a locked cabinet in an area with limited access. All participant information will be stored in a separate locked cabinet.

### Ethics and dissemination

This protocol has been approved by the Medical Ethics Committees at Tianjin University of Traditional Chinese Medicine (TJUTCM-EC20170004). Written and informed consent will be fully explained by the acupuncturist and signed by the participants before entering the trial. The protocol has been registered at ClinicalTrials.gov. Any modification of the protocol will be documented at ClinicalTrials.gov. The results of this study will be published in a peer-reviewed journal.

## Discussion

BCRL is a common complication after breast cancer treatment despite the application of less invasive surgical techniques and BCRL patients often suffer from severe physical and psychological morbidity. Patients are in constant need of an effective treatment to manage lymphedema [21]. Conservative intervention such as complete decongestive therapy (CDT) is often considered as the first-line intervention and different studies have shown that CDT may be able to reduce lymphedema volume of the affected limb [22]. However, CDT is an intensive treatment program that requires daily one-on-one treatment for 4–6 weeks with a specialized therapist [23]. Therefore, it is often considered costly and time-consuming [24, 25]. In addition, long-term use of compression garments is required to maintain the initial limb-volume reduction [26], which results in significant inconvenience and discomfort for the patients.

According to several pilot studies, acupuncture emerges as a potential treatment option given it convenient treatment modality with very few side effects. Our preliminary study also found satisfactory results with sustained effect up to 1-month follow-up. Acupuncture is also more convenient and less expensive when compared to CDT. However, since lymphedema is a rather modern disease, the most effective acupuncture prescription has not been established. Indeed, a thorough search of the PubMed database showed a variety of acupoint prescriptions for the treatment of BCRL – one study [9] with flexible acupuncture points, one study [5] with acupuncture points on the affected limb only (local distribution acupoint combination), two studies [6, 7] with distal acupuncture points only (distal distribution acupoint combination), and two studies [8, 10] with whole body acupuncture points (local-distal acupoint combination). Therefore, additional research is required to compare the effectiveness of different acupuncture point combinations.

In this study, we will compare the two classic methods for combining acupuncture points – local distribution combination and local-distal combination. A local distribution combination is defined as local acupoints on the affected arm where the symptoms manifest and a local-distal combination is defined as acupoints on the affected arm combined with acupoints distant from the affected arm such as on the abdomen, unaffected arm, or legs. In clinical practice, a local-distal combination is the most commonly used treatment prescription, but a local distribution combination can be very effective when targeting a local symptom. By comparing the two methods, we will be able to gain knowledge of general principles in treating BCRL, which will then serve as a fundamental basis for future refinement of points prescription. In both acupuncture groups, we will integrate needle-top moxibustion to the treatment plan since moxibustion can resolve fluids according to traditional Chinese medicine theories. An observational study by Li et al. [27] also showed that thermotherapy was able to reduce swelling and improve quality of life. Therefore, acupuncture combined with needle-top moxibustion (warm acupuncture) may be more effective in managing BCRL.

There are some limitations in this study. First, the follow-up period is only 1 month. A longer follow-up period will allow us to assess the long-term effect of acupuncture treatments. Second, the amount of moxibustion applied is different in the LA and LDA groups. The probable compounding effect of greater moxibustion application and the local-distal acupoint combination in the LDA group may result in increased therapeutic dosage in that group and thus interfere with the comparison between local distribution acupoint combination and local-distal acupoint combination.

In conclusion, this is a rigorous, larger, multi-center, randomized controlled trial, which will enable us to further assess the effectiveness of acupuncture in the treatment of BCRL and also compare the effectiveness between local distribution and local-distal acupoint combinations.

## Trial status

Protocol version number: 1.2 (2017/12/5)

Date recruitment began: 2018/1/19

Date when recruitment will be completed: 2019/5/1

## Additional file

Additional file 1: SPIRIT 2013 Checklist: recommended items to address in a clinical trial protocol and related documents*. (DOC 124 kb)

## Data Availability

Data supporting the findings of this study are available from the corresponding author.

## Abbreviations

BCRL: Breast cancer-related lymphedema
CDT: Complete decongestive therapy
CRF: Case report form
CTCAE: Common Terminology Criteria for Adverse Events
DASH: Disabilities of the Arm, Shoulder, and Hand
SF-36: Medical Outcome Study 36-item Short-Form Health Survey
